# Heightened response to positive facial cues as a potential marker of resilience following childhood adversity

**DOI:** 10.1080/20008066.2024.2309783

**Published:** 2024-02-06

**Authors:** Mattia I. Gerin, Essi Viding, Louise Neil, Diana J. N. Armbruster-Genc, Ze Freeman, Molly Sharp, Harriet Phillips, Eamon J. McCrory

**Affiliations:** a Division of Psychology and Language Sciences, University College London, London, UK; b Anna Freud National Centre for Children and Families, London, UK

**Keywords:** Maltreatment, child abuse, early adversity, emotion intensity, positive affect, resilience, social support, Maltrato, abuso infantil, adversidad temprana, intensidad emocional, afecto positivo, resiliencia, apoyo social

## Abstract

**Background:** Childhood maltreatment profoundly influences social and emotional development, increasing psychiatric risk. Alterations in the implicit processing of threat-related cues following early abuse and neglect represent a marker of mental health vulnerability. Less is known about how early adversity influences the perception of positive social cues, despite their central role in establishing and maintaining social interactions and their association with better mental health outcomes.

**Methods:** The sample consisted of 42 children and adolescents with substantiated childhood maltreatment experiences and 32 peers (mean age 13.3), matched on age, pubertal status, gender, socioeconomic status, ethnicity, and cognitive ability. A computerised experimental task assessed the perceived emotional intensity of positive (happy) and negative (fearful) facial expressions. Mental health symptoms were measured via self- and parental reports, and perceived social support was self-reported.

**Results:** The experience of abuse and neglect was associated with heightened perceived intensity of positive facial cues. Cross-sectional post-hoc moderation and mediation analyses, employing a model-building approach, revealed that in maltreatment-exposed participants: (i) their increased response to positive facial cues was associated with lower symptoms; (ii) the presence of social support accounted for their heightened perceived intensity of positive facial cues; (iii) the presence of social support putatively contributed to lower symptoms by increasing the perceived intensity of positive facial cues. No group differences in perceived intensity of negative expressions were observed.

**Conclusions:** These findings provide fresh insight into how positive faces are processed following maltreatment experience in childhood. Maltreatment experience was found to be associated with heightened perceived intensity of happy faces, which in turn was associated with better mental health and greater levels of social support. This suggests that heightened saliency of positive emotions acts protectively in children with maltreatment experience.

## HIGHLIGHTS

- The impact of childhood maltreatment on perceiving positive social cues, especially explicit emotional intensity appraisal, is less understood compared to the extensive focus on its role in processing threat-related cues and psychiatric risk.
- In this study, we found that maltreatment experience was associated with heightened perceived intensity of happy faces, which in turn was associated with better mental health and greater levels of social support.
- These findings suggest that heightened saliency of positive emotions may act protectively in children with maltreatment experience.

## Abstract (es)

**Antecedentes:** El maltrato infantil influye profundamente en el desarrollo social y emocional, incrementando el riesgo de enfermedades psiquiátricas. Las alteraciones en el procesamiento implícito de señales de amenaza tras el abuso y negligencia tempranas representan un indicador de vulnerabilidad en la salud mental. Poco se sabe sobre cómo la adversidad temprana afecta la percepción de señales sociales positivas, a pesar de su rol central en el establecimiento y mantenimiento de interacciones sociales y su asociación con resultados de salud mental más favorables.

**Método:** La muestra incluyó a 42 niños y adolescentes con experiencias de maltrato infantil corroboradas y 32 pares (Edad media 13,3 años), emparejados por edad, etapa puberal, género, nivel socioeconómico, etnia, y capacidad cognitiva. Un modelo computarizado, evaluó la percepción de la intensidad emocional positiva (felicidad/alegres) y negativa (temerosa) de las expresiones faciales. Los síntomas de salud mental fueron medidos a través de reportes propios y de los padres, y el apoyo social percibido fue autoreportado.

**Resultados:** Las experiencias de abuso y negligencia se asociaron con una mayor percepción de intensidad de las señales faciales positivas. El análisis de moderación y mediación post-hoc (o análisis derivados de datos) de corte transversal, utilizando un enfoque de construcción de modelos, reveló que en los participantes expuestos al maltrato: (i) su mayor respuesta a las señales faciales positivas estaba asociada a baja sintomatología; (ii) la presencia de apoyo social explicó la mayor intensidad percibida de las señales faciales positivas; (iii) la presencia de apoyo social contribuía supuestamente a reducir sintomatología al aumentar las señales faciales positivas percibidas. No se observaron diferencias en la intensidad percibida de las expresiones negativas entre los grupos.

**Conclusiones:** Estos hallazgos aportan una nueva visión sobre cómo las expresiones de caras positivas son procesadas, posterior a experiencias de maltrato durante la infancia. Se encontró que la experiencia de maltrato está asociada con la percepción de mayor intensidad de las caras felices, lo que a su vez se asoció con una mejor salud mental, y mayores niveles de apoyo social. Esto sugiere que el aumento de la intensidad de las emociones positivas actúa como un efecto protector en niños con experiencias de maltrato.

## 1. Introduction

Childhood maltreatment – any act or series of acts of commission or omission by a parent or other caregiver that results in harm or potential for harm – is robustly associated with an increased likelihood of enduring behavioural and psychological difficulties (Gilbert et al., 2009; Strathearn et al., 2020) and with a reduced response to currently available treatments (Nanni et al., 2012; Nelson et al., 2017; Teicher et al., 2022). A growing body of research suggests that increased psychiatric risk following experiences of abuse and neglect is linked with alterations in cognitive and neurobiological functioning (McCrory et al., 2017; McLaughlin & Lambert, 2017; Teicher & Samson, 2016). These include changes in learning, reward processing, decision-making, and autobiographical memory (Armbruster-Genç et al., 2022; Gerin et al., 2017; McCrory et al., 2017; Valentino et al., 2009), as well as shifts in attention and emotional processing, such as threat detection, emotion recognition, and regulation (Gerin et al., 2019; Kim & Cicchetti, 2010; McCrory et al., 2011; Pollak & Sinha, 2002; Weissman et al., 2019). Maltreatment exposure has also consistently been associated with the altered allocation of cognitive resources to threat-related visual cues. This includes greater attentional capture and heightened recognition of fearful or angry facial expression, and increased neural response to threat-related cues. These findings have invariably been reported during implicit processing conditions – that is, when stimuli have been presented incidentally, under conditions of cognitive load or pre-attentively (McCrory & Viding, 2015). On the other hand, the impact of childhood adversity on emotional processing for positive emotions is less consistent (English et al., 2018; Kelly et al., 2015; McCrory et al., 2011; McCrory et al., 2013; Pine et al., 2005; Pollak et al., 2009; Pollak & Tolley-Schell, 2003).

The neurocognitive social transactional model by McCrory and colleagues (McCrory et al., 2022) postulates that neurocognitive alterations following maltreatment exposure impact (in a bidirectional and iterative fashion) an individual’s social interactions in ways that potentiate (or reduce) psychiatric risk. Specifically, it has been suggested that maladaptive (or protective) neurocognitive profiles influence the social architecture individuals construct around themselves across development. Conversely, an individual’s social ecology can either contribute to ongoing poor optimisation of neurocognitive development or, act protectively by facilitating recalibration or compensation in neurocognitive development. For example, it has long been known that individuals exposed to early adversity experience more peer rejection, victimisation, and interpersonal conflict, or greater ‘stress generation’ (Bolger & Patterson, 2001; McCrory et al., 2022; Widom et al., 2014). They also experience a reduction in the extent and quality of supportive relationships over time, ­a phenomenon that has recently been termed ‘social thinning’ (McCrory et al., 2022; Sperry & Widom, 2013). Extant data indicate that stress generation and social thinning after maltreatment exposure predict future psychopathology (Gerin et al., 2019; Sperry & Widom, 2013). In other words, across the life course, transactional processes between the individual and their social environment unfold in ways that can increase, or decrease, the risk of psychopathology (McCrory et al., 2022). Currently, we still have limited understanding of the range of neurocognitive processes that may be associated with social functioning and mental health outcomes in individuals with maltreatment experience. Alterations in affective processing, already implicated in maltreatment exposure, offer a promising avenue for further exploration in this area, given that emotions are intricately linked to social information-processing and mental health (Eisenberg et al., 2000; Halberstadt et al., 2001; Lemerise & Arsenio, 2000).

The way emotions are experienced and perceived can influence an individual’s sense of connectedness with others (Mauss et al., 2011; Mei et al., 2018; Oosterhof & Todorov, 2009; Van Boven et al., 2010) and is associated with su ccessful interpersonal problem-solving (Williams et al., 2005). The saliency (i.e. the perceived importance or prominence of emotional stimuli) attributed to positive affect is critical to resilient functioning in heightening the attentional capture of affiliative cues (Taylor et al., 2010; Thoern et al., 2016; Troller-Renfree et al., 2017). Consistent with this concept, various domains of emotion processing, including perception, regulation, and expression, have been shown to predict social functioning among children not selected based on maltreatment status (Eisenberg et al., 1995; Eisenberg et al., 1997) as well as among young people who experienced abuse or neglect (Rogosch et al., 1995).

The ability to evaluate the intensity of others’ affective cues – that is, the explicit appraisal of the perceived magnitude of an emotional expression – is crucial to mental health and social functioning (Cole et al., 2004; Reisenzein, 1994). In non-clinical samples, neurophysiological data indicate that people with high self-esteem are more responsive to positive facial expressions and less reactive to negative ones than those with low self-esteem (Wang & Wu, 2019). Also, the perceived intensity of emotions has been shown to influence essential domains of social cognition, such as perceived trustworthiness (Engell et al., 2010; Oosterhof & Todorov, 2009) and psychological closeness/distance (Mei et al., 2018; Van Boven et al., 2010). Among clinically and sub-clinically depressed individuals, positive affect, such as happy facial expressions, is perceived as less intense (Dai & Feng, 2012; Yoon et al., 2009); while negative affect, such as sad or angry facial expressions, is rated as more intense (Branco et al., 2018; Dai & Feng, 2012; Naranjo et al., 2011; Yi et al., 2016). These findings indicate that the perceived intensity of others’ emotions may influence affiliative behaviours in ways that contribute to better or worse mental health outcomes and may also contribute to therapeutic rapport (and thus influencing treatment response) (Sharpley et al., 2006). This is consistent with established findings that atypical cognitive and emotional processing contributes to the emergence and maintenance of psychopathology by shaping an individual’s social experience (Hammen, 2006; Hirschfeld et al., 2000; Snyder-Mackler et al., 2020).

### 1.1. Aims and hypotheses

Previous research on emotional processing in young people exposed to childhood maltreatment has primarily focused on aspects like emotion recognition and attentional capture. In these domains, there is strong evidence for altered implicit processing of negative emotions and mixed evidence for positive emotions (Hart et al., 2018; McCrory et al., 2013; Pollak & Kistler, 2002; Pollak & Sinha, 2002). However, no prior study has assessed whether maltreatment experience is associated with differences in the *explicit appraisal of emotional intensity*. In this context, ‘explicit appraisal’ refers to the conscious and deliberate evaluation of the intensity of emotional cues. This form of appraisal reflects an individual's subjective, consciously-aware assessment of others’ affective signals and has been linked to psychosocial and mental health outcomes (Cole et al., 2004; Dai & Feng, 2012; Engell et al., 2010; Mei et al., 2018; Oosterhof & Todorov, 2009; Reisenzein, 1994; Van Boven et al., 2010; Wang & Wu, 2019; Yoon et al., 2009).

Our study had two main aims. First, considering evidence linking the perception of emotional intensity to mental health outcomes, we sought to experimentally investigate the relationship between prior substantiated maltreatment experience and the perceived explicit intensity of others’ emotions. We employed dynamic facial stimuli of happiness and fear to increase ecological validity. In light of the evidence of increased saliency and neural reactivity for both positive and negative social cues following abuse and neglect (Dennison et al., 2016; Hart et al., 2018; McCrory et al., 2013), we predicted greater intensity ratings for both happy and fearful faces in adolescents with maltreatment experience.

Second, using a range of measures assessing psychological and behavioural functioning, we aimed to explore whether differences in the perceived intensity of others’ emotions were associated with mental health symptoms and perceived degree of social support. Heightened neural reactivity to negative social stimuli in young people exposed to childhood maltreatment predicts future increased internalising symptoms (Gerin et al., 2019), while heightened neural reactivity to positive social stimuli among adolescents exposed to childhood abuse is prognostic of fewer internalising symptoms (Dennison et al., 2016). In light of these findings, we predicted that psychological and behavioural problems following maltreatment experience would be mediated by perceived intensity of reward-related facial cues (happiness) – with reduced problems associated with greater perceived intensity. We also predicted that higher levels of psychological and behavioural difficulties would be mediated by heightened perceived intensity of threat-related facial cues (fear). In addition, we sought to explore whether the presence of social support following maltreatment exposure would act as a protective factor, partly accounting for (i.e. moderate) heightened perceived intensity of positive affect, and, in turn, putatively contribute to fewer mental health symptoms.

## 2. Methods

### 2.1. Participants

Seventy-six children and adolescents (mean age = 13.3) participated in this study (Table 1). Forty-two participants who had experienced maltreatment requiring social services intervention (maltreatment group, MT) were recruited via London Social Services departments. The parent, legal guardian and/or social work professional were asked to assess current safety and stability of placement before providing consent for any child or young person to participate. Thirty-four peers with no prior Social Services contact (non-maltreatment group, NMT) were recruited through schools in similar areas to match the MT group on demographic variables, including socioeconomic status, ethnicity, age, pubertal status, sex, and IQ. Exclusion criteria included a pervasive developmental disorder (e.g. a diagnosed learning disability, autistic spectrum disorder), neurological abnormalities, and an IQ below 70. Following the outlier removal procedure based on Tukey’s box-plot inter-quartile range using a multiplier of 2.2 (Hoaglin & Iglewicz, 1987), 2 NMTs were removed. The final sample consisted of MT = 42 and NMT = 32 (total *N* =  74). The participants’ parents or legal guardians provided written consent, and all participants provided written assent. The UCL Research Ethics Committee approved the study (11767/001).

**Table 1.** Demographics, Cognitive Abilities, Psychological, Behavioural and Social Functioning in the Maltreated (MT; *n* = 42) and non-maltreated group (NMT; *n* = 32).

	MT	NMT
Measures	Percentage	Percentage
Female	52.4%	56.3%
Caucasian	40.5%	40.6%
	Mean (SD)	Mean (SD)
Age^a^	13.3 (2.1)	13.2 (1.9)
PDS^b^	2.5 (0.8)	2.4 (0.8)
WASI-IQ^c^	100.2 (12.4)	104.8 (12.4)
SES^d^	3.3 (1.0)	2.9
SDQ total score^e^ ***	12.9 (5.5)	8.9 (4.3)
CASSS^f^	58.8 (8.0)	56.2 (11.8)

Notes: *** *p* < .001.

^a^ age range = 9–16 years. ^b^PDS = Puberty Development Scale (Petersen et al., 1988), a composite score of parent and child reports (in each group, the parent report was not available for one participant, and the child report was not available for two participants). ^c^WASI-IQ = two IQ-subscales derived from the Wechsler Abbreviated Scales of Intelligence (Wechsler, 2011). ^d^SES = socioeconomic status; highest parental/carer level of education rated on a 6-point scale from 0 (no formal qualifications) to 5 (postgraduate qualifications). ^e^SDQ = Strength and Difficulties Questionnaire (Goodman, 2001), a composite score of parent and child reports (for one NMT participant the parent report was not available). ^f^CASSS = total frequency scores of the close friend subscale of the Childhood and Adolescent Social Support Scale; MT = 40, NMT = 31 with available CASSS data (Malecki & Demaray, 2002).

### 2.2. Measures

**Maltreatment History.** The presence of maltreatment experience was based on Social Services reports. The severity of each maltreatment subtype, using Kaufman’s scale (Kaufman et al., 1994), was rated on a scale from zero (not present) to four (Supplementary Information, Table S1). The frequency of each maltreatment category included neglect = 66.7%, emotional maltreatment = 90.5%, sexual abuse = 9.5%, and home violence = 85.7%. Within the MT group, 4.8% experienced one, 40.5% experienced two, 50% experienced three, and 4.8% experienced four childhood maltreatment subtypes.

**Psychological, Behavioural and Social Functioning Measures.** Parent and child versions of the Strengths and Difficulties Questionnaire (SDQ) were administered to assess psychological and behavioural difficulties (Goodman, 2001). The SDQ assess four symptom domains – emotional symptoms, conduct problems, hyperactivity/inattention, and peer relationships problems – which are added together to generate a total difficulties score. The parent- and child-reported scores were averaged to create a combined total difficulties score (Table 1). The SDQ is characterised by sound internal consistency (Cronbach alpha = .73), and test-retest reliability (*r* = 0.62). The child-reported ‘close friend’ subscale of the Child and Adolescent Social Support Scale (CASSS) was administered to assess perceived peer social support, an essential social functioning domain for children and young people (Malecki & Demaray, 2002). The close friend subscale has sound internal consistency (Cronbach alpha across age groups ranges from .93 to .97.) and test-retest reliability (*r* = 0.70).

**Emotional Intensity.** A computerised experimental task was used to examine the perceived emotional intensity of facial expressions of fear and happiness. Participants were presented with dynamic facial stimuli from the Amsterdam Dynamic Facial Expression Set – Bath Intensity Variations (ADFES-BIV) (Wingenbach et al., 2016). This validated set of videos portrays three intensity levels of emotional expressions – low, medium, and high. Each stimulus, which lasts 1040ms, begins with a neutral expression and continues until the end of the expression. After each stimulus, participants identified the emotion presented from a forced choice of the five (joy, sadness, surprise, fear, and neutral) and then rated the intensity of the expression from 1 (very little) to 10 (a lot). For faces participants identified as neutral, no intensity rating was requested. Participants initially completed five practice trials (one for each facial expression type). Then they were randomly presented with 24 trials of happy and 24 trials of fearful facial expressions (eight for each intensity level). To reduce a tendency to perseverate, stimuli of no interest were also randomly presented, comprising fourteen ‘filler’ trials, including eight neutral, three sad (one for each intensity level), and three surprise (one for each intensity level) facial expressions. A mean intensity score for all correctly identified positive faces (i.e. happy faces) and one for all negative faces (i.e. fear faces) was calculated and used in the statistical analyses. Filler trials were not analysed.

### 2.3. Procedures

Demographic information, symptoms questionnaire and psychometric testing were completed by the participant and one parent/carer during an initial home visit. Some participants completed the emotional intensity task during the same home visit, while others attended a second session at University College London (UCL) campus. The session on campus also involved other behavioural experiments and, for some participants, a brain scan.

### 2.4. Data analysis

Independent sample *t*-tests and chi-squared tests were performed to explore whether the MT and NMT groups differed on background characteristics – demographics, cognitive abilities, psychological, behavioural and social functioning (Table 1). A two-way (2×2) mixed design ANOVA, with maltreatment status (i.e. the independent variable) as a between-subjects factor and mean emotional intensity scores (for fearful and happy faces – i.e. the dependent variable) as a within-subjects factor, was performed to examine the hypothesised group differences in perceived emotional intensity. We also carried out a number of exploratory post-hoc analyses. Sensitivity analyses were performed by removing participants who met the clinical threshold on the SDQ total score – this was done to assure that results were not driven by participants who present a frank mental health difficulty. We also performed t-tests to explore if any group difference was driven by low, medium or high-intensity facial expressions. Finally, mediation, moderation, and mediated-moderation models, implemented in SPSS (v.28) using the software package PROCESS (v4.1), were used for post-hoc explorations of the associations between maltreatment status, emotional intensity scores, mental health symptoms and social support (Hayes, 2017). Two participants in the MT group and one participant in the NMT group did not have available CASS data – these were excluded from the moderation and mediated moderation analyses.

## 3. Results

Table 1 presents an overview of descriptive and inferential statistics related to the group's demographics, cognitive abilities, psychological, and social functioning.

### 3.1. Childhood maltreatment and perceived emotional intensity

A mixed design ANOVA revealed a statistically significant main effect of emotion [*F*(1,72) = 30.77, *p <* .001, *η_p_^2^ = *.30], such that across groups fearful faces, *M(SD)* = 5.7(1.4), were rated as more intense than happy faces, *M(SD)* = 5.0(1.2). The main effect of group was non-significant [*F*(1,72) = .64, *p =* .43, *η_p_^2^ = *.01]. However, the interaction between emotion and group was significant [*F*(1,72) = 7.26, *p <* .01, *η_p_^2^ = *.09]. Follow-up *t*-tests analyses showed that the MT group rated happy faces as more intense than the NMT group [*M(SD)_MT _*= 5.3(1.4); *M(SD)_NMT _*= 4.7(0.9); *t*(70.04) = 2.10, *p* = .02, *d*’ = 0.46], while the two groups did not differ on how intensely they rated fearful faces [*M(SD)_MT _*= 5.6(1.5); *M(SD)_NMT _*= 5.7(1.3); *t*(72) = 0.34, *p* = .37, *d*’ = 0.08].

**Figure 1.**
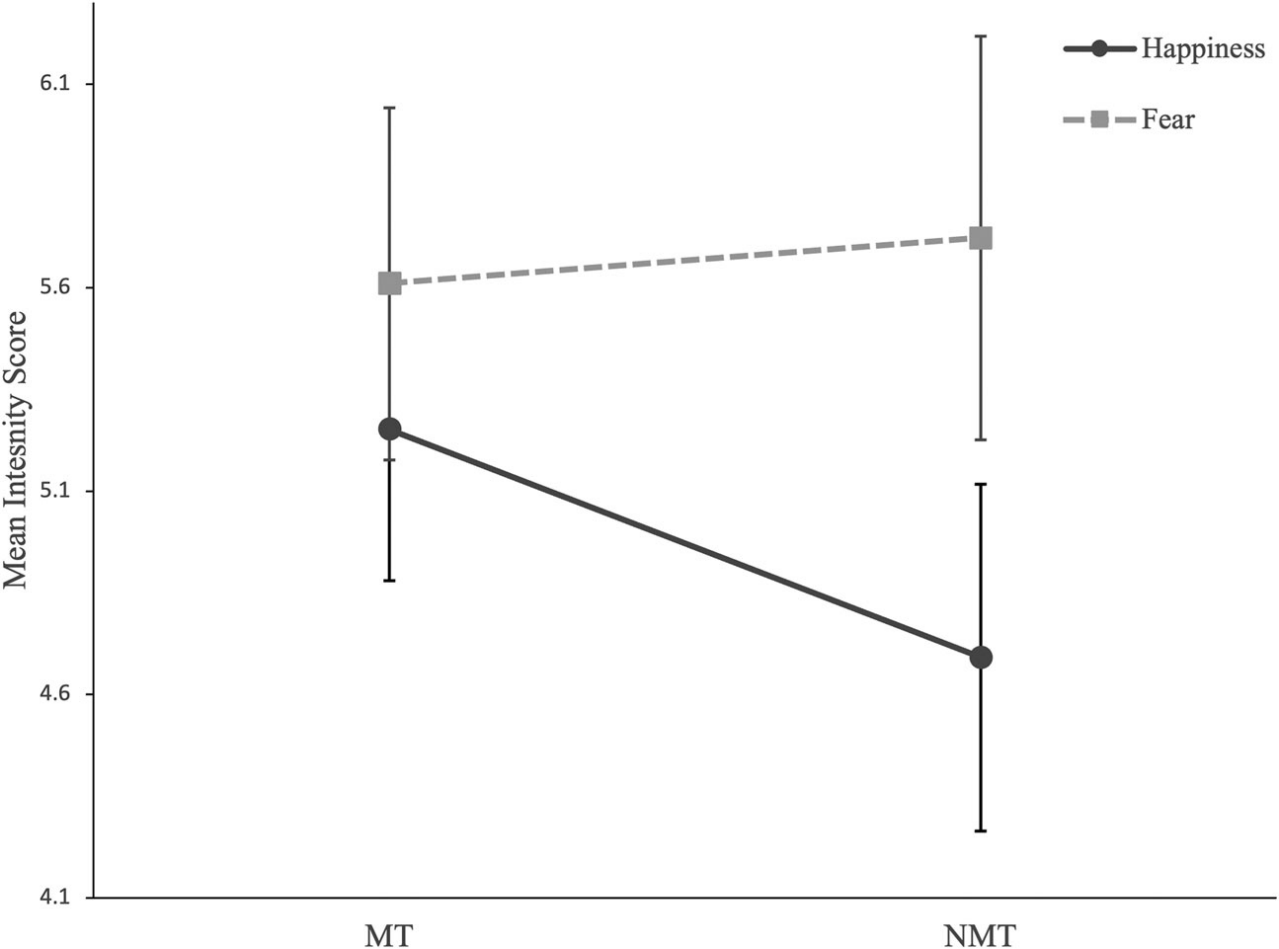
Mean Intensity Scores for Happy and Fearful Facial Expressions in the MT and NMT. Note. Error bars represent 95% confidence intervals.

Sensitivity analyses revealed that the main effect of emotion [*F*(1,63) = 20.80, *p <* .001, *η_p_^2^ = *.25] and the interaction effect between group and emotion [*F*(1,63) = 10.22, *p <* .01, *η_p_^2^ = *.14] remained significant after removing participants who met the clinical threshold on the SDQ total score (i.e. SDQ total scores equal or above 17; remaining sample: MT = 34 and NMT = 31). Also, as reported in greater detail in the Supplementary Information document, the groups did not differ in their ability to recognise and correctly label positive and negative facial expressions [*F*(1,72) = 1.00, *p =* .32, *η_p_^2^ = *.01] (Figure 1).

### 3.2. Post-hoc analyses

**Low, Medium and High-Intensity Happy Faces.** Post-hoc analyses (using a Bonferroni adjusted alpha level of .017) revealed that the group difference in happiness intensity scores was primarily driven by medium-intensity happy facial stimuli [*M(SD)_MT _*= 4.6(1.5); *M(SD)_NMT _*= 3.9(0.9); *t*(69.29) = 2.36, *p* = .01, *d’ = 0.52*]*,* and approached statistical significance for low-intensity happy facial stimuli [*M(SD)_MT _*= 3.3(1.8); *M(SD)_NMT _*= 2.6(1.2); *t*(70.62) = 1.87, *p* = .03, *d’ = 0.41*]*.* Groups’ ratings did not differ for high-intensity happy facial stimuli [*M(SD)_MT _*= 6.9(1.3); *M(SD)_NMT _*= 6.7(1.1); *t*(72) = 0.75, *p* = .23, *d’ = 0.18*].

**Figure 2.**
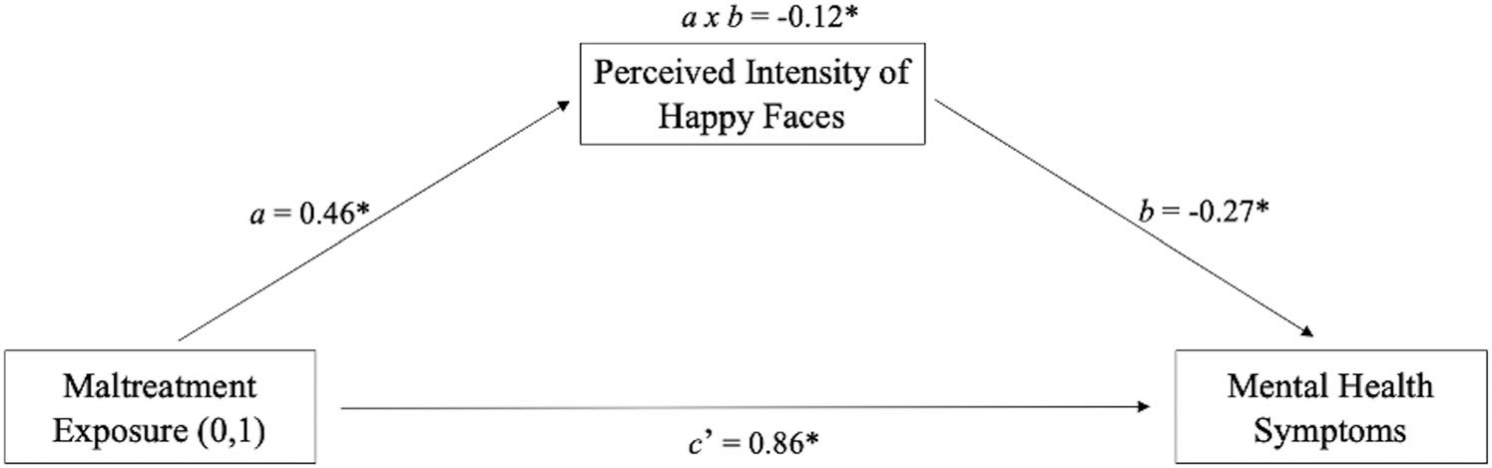
A Mediation Model Depicting the Associations Between Maltreatment Exposure (MT and NMT groups), Mean Intensity Scores for Happy Faces, and Mental Health Symptoms (SDQ Total Score). Note. Coefficient values are standardised; the interaction term (i.e. indirect effect: *a x b*) significance threshold is measured using bootstrapping (*n* = 5000, CI = 95%), and heteroscedasticity consistent Huber-White standard error is implemented; *N* = 74 (MT = 42; NMT = 32); * = statistically significant coefficients.

**Childhood Maltreatment, Perceived Emotional Intensity, and Behavioural and Psychological Functioning.** A cross-sectional mediation analysis was performed to investigate if the perceived emotional intensity of happy faces (i.e. the mediator) following early adversity (i.e. the independent variable) was associated with the severity of psychological and behavioural symptoms (i.e. the dependent variable), as captured by the combined parent and child-rated SDQ total score. As shown in Figure 2, maltreatment is associated with overall higher presenting symptoms (pathway c’), and a statistically significant partial indirect pathway (*a x b*) indicates that heightened perceived intensity of happy facial expressions following maltreatment exposure is associated with better behavioural and psychological functioning (i.e. lower overall presenting symptoms). That is, perceiving positive facial expressions as more intense following maltreatment exposure was associated with fewer symptoms.

**Figure 3.**
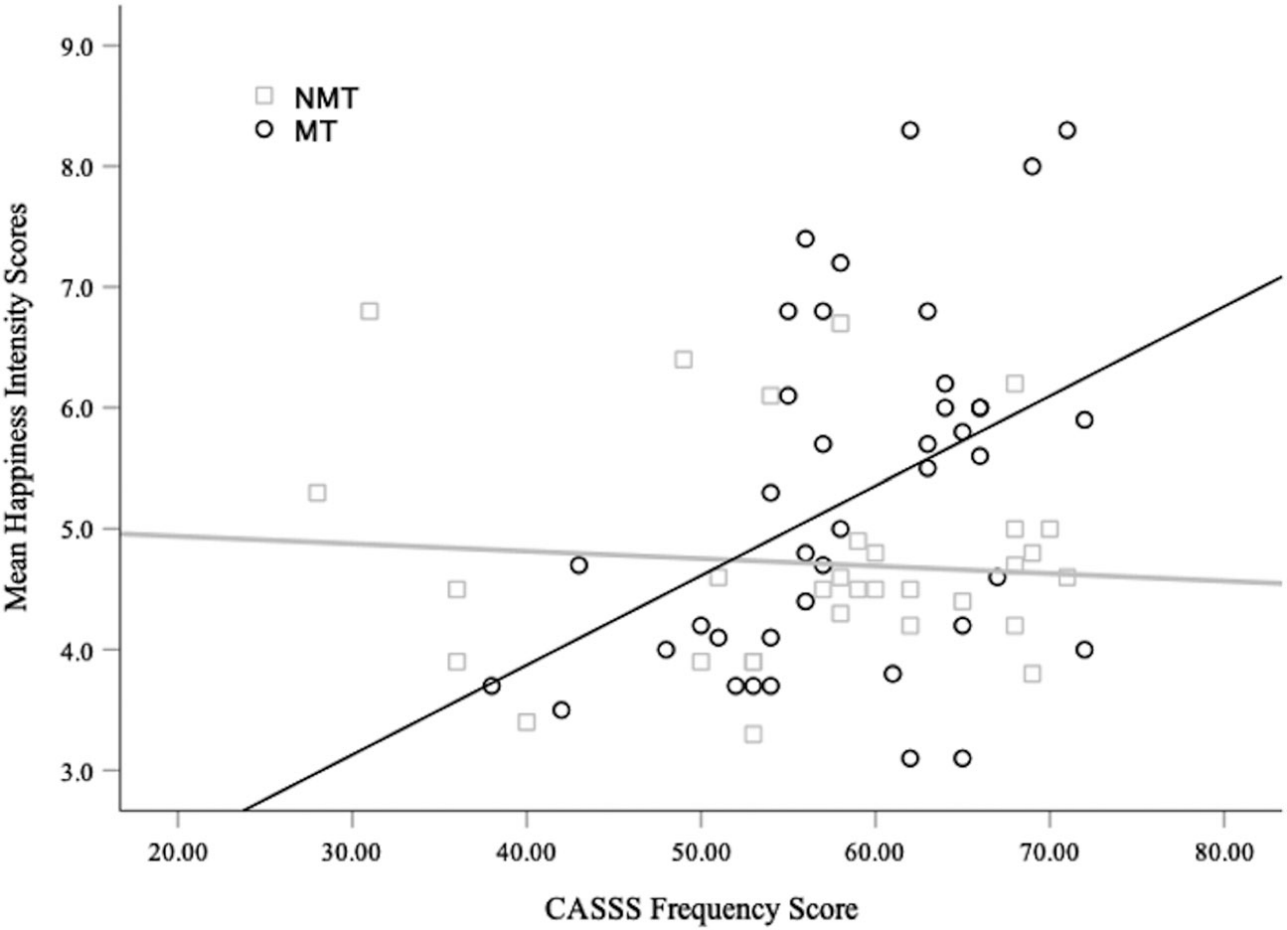
Mean Intensity Scores for Happy Faces are Plotted as a Function of Social Support (CASSS Frequency Scores) and Maltreatment Status (MT and NMT groups).

**Childhood Maltreatment, Social Support, and Perceived Emotional Intensity.** A cross-sectional moderation model was performed to investigate whether the association between maltreatment exposure and emotional intensity scores for happy faces was related to the amount of perceived social support from peers (i.e. CASSS frequency scores). We found that social support moderated the association between maltreatment status (the independent variable) and emotional intensity ratings for happy faces (the dependent variable); that is, the interaction term (*maltreatment group status x CASSS frequency score*) was found to be significant *β_standardised_* = .63, *t*(67) = 2.94, *p* > .01. As depicted in Figure 3, maltreatment exposure was associated with increased perceived emotional intensity of happy faces only among those who had higher levels of social support. The Supplementary Information (Table S3) reports the full regression model.

**Figure 4.**
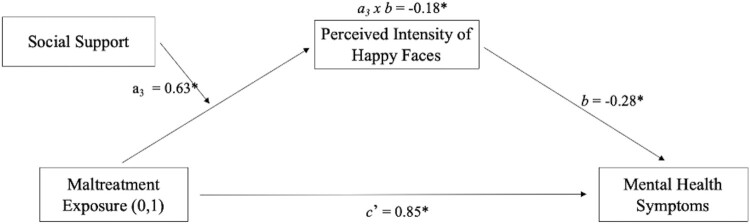
A Moderated-Mediation Model Depicting the Associations Between Maltreatment Exposure (MT and NMT groups), Social Support (CASSS Frequency Scores), Mean Intensity Scores for Happy Faces, and Mental Health Symptoms (SDQ Total Score). Note. Coefficient values are standardised; the moderated-mediation coefficient (i.e. conditional indirect effect: a_3_ x b) significance threshold is measured using bootstrapping (*n* = 5000, CI = 95%), and a heteroscedasticity consistent Huber-White standard error is implemented; *N* = 71 (MT = 40; NMT = 31); * = statistically significant coefficients.

**Childhood Maltreatment, Social Support, Perceived Emotional Intensity, and Behavioural and Psychological Functioning.** All the variables of interest were included in one cross-sectional moderated-mediation model (in which the ‘a’ pathway is moderated) to examine whether the increased perceived emotional intensity of happy faces following early adversity and its association with symptom severity depended on the amount of perceived social support from peers (Figure 4). The significant index of moderated-mediation (pathway a_3_ x b; Figure 4) indicates the presence of an indirect conditional effect. Social support (i.e. CASSS scores) was found to moderate the association between maltreatment exposure (i.e. the independent variable) and symptoms (i.e. SDQ total score – the dependent variable) via emotional intensity scores for happy faces (i.e. the mediator). That is, the model suggests that following maltreatment exposure during childhood, the presence of higher levels of social support from peers may be linked with resilient psychological and behavioural outcomes by heightening the perceived intensity of positive affect. As reported in greater detail in the Supplementary Information document, when performing the same moderated-mediation model by swapping, in all possible combinations, the dependent, moderating, and mediating variables (i.e. social support, symptoms severity and emotional intensity scores for happy faces), the indexes of moderated-mediation in all models were non-significant. This provides tentative evidence, to be confirmed in future longitudinal studies, that the indirect conditional effect reported in Figure 4 may be directional.

## 4. Discussion

This study examined if children and adolescents with maltreatment experience show atypical *explicit* appraisal of facial affect. Consistent with our predictions, independent of clinical status and demographic confounders, children and adolescents with a history of maltreatment showed heightened perceived intensity of happy faces compared to non-exposed peers (Figure 1). This was driven by intensity appraisals of more subtle low- and medium-intensity happy faces rather than appraisals of unambiguously positive faces. These findings are in line with previous studies showing that early adversity influences emotional development – including alterations in emotion recognition, emotion regulation, and neural response to affective cues (Gerin et al., 2019; Kim & Cicchetti, 2010; Pollak et al., 2000). They also extend our knowledge about the impact of abuse and neglect on the perception of positive affect and *explicit* emotional processing – most studies on positive affect had focused on pre-attentive and incidental processing of affective cues (Dennison et al., 2016; McCrory et al., 2013).

Contrary to predictions, there was no group difference in the intensity ratings of fearful faces. This finding suggests that altered processing of threat-related cues following maltreatment exposure, may be primarily manifest under conditions of implicit processing (e.g. cognitive load, incidental and pre-attentive perception) (English et al., 2018; Kelly et al., 2015; McCrory et al., 2013), rather than, as was the case in this study, during explicit processing and the declarative appraisal of affect. It is important to note that our study did not directly compare implicit and explicit processing. Therefore, the implications regarding the implicit versus explicit processing of threat-related social cues should be considered tentatively, and further research is needed to explore this.

Using a model-building analytic approach the group difference in the perceived intensity of positive facial stimuli was further explored with cross-sectional mediation and moderation analyses. First, a mediation model showed that assigning greater emotional intensity to positive social cues partly accounted for lower presenting symptoms among children and adolescents with maltreatment experience (Figure 2). This finding aligns with maltreatment and clinical literature that has found that increased salience of positive affect is associated with resilient outcomes. For example, increased neural responsiveness to positive social stimuli after maltreatment is a longitudinal predictor of lower internalising symptoms (Dennison et al., 2016). Also, increased saliency of happy facial expressions is more prevalent among individuals with lower internalising symptoms and higher self-esteem, a critical aspect of effective interpersonal functioning and mental health (Wang & Wu, 2019; Yoon et al., 2009). Second, moderation and moderated-mediation models indicated that the presence of higher levels of social support from peers accounted for the heightened perceived intensity of happy faces among maltreatment-exposed participants (Figure 3), which, in turn, mitigated the association between maltreatment exposure and higher presenting mental health symptoms (Figure 4). These results are consistent with the evidence that following maltreatment exposure, an individual’s social architecture and interpersonal experiences (e.g. social support, stable foster placements, loneliness, and victimisation from peers) are important for psychological and behavioural outcomes (Goemans et al., 2021; McCrory et al., 2022; Sperry & Widom, 2013). In particular, they suggest that the presence of social support following early adversity may lead to protective recalibrations in the subjective appraisal of reward-related social stimuli.

For individuals who grow up in neglectful and abusive households – characterised by less frequent and predictable social reinforcers, such as verbal praise, physical affection, and smiles (Wilson et al., 2008) – assigning greater saliency to more subtle positive social cues may represent an adaptive trait. Being more sensitive to, and maximising infrequent or inconstant predictors of, rewards may increase the likelihood of obtaining such rewards, experiencing greater interpersonal self-efficacy and positive emotions. The latter, according to Fredrickson's ‘Broaden-and-Build’ theory, is believed to significantly promote resilient mental health outcomes (Fredrickson, 2000; Fredrickson, 2001). According to this theoretical framework, positive emotions are not just an outcome of adaptative behaviour, but also a precursor of it (Fredrickson, 2001). The experience of positive affect – such as happiness, interest, anticipation, and contentment – is thought to *broaden* momentary attentional resources, as well as an individual’s behavioural and thinking repertoire (Fredrickson, 2001). This expanded range of actions and cognitions, over time, can enable the *building* of adaptative skills and psychological resources that persist beyond the positive affect through which they were initially acquired. For instance, it is thought that positive emotions, such as curiosity, can facilitate exploratory behaviour and learning. Experiencing positive emotions can also facilitate pleasant interactions with others, which, over time, can lead to the formation of meaningful interpersonal connections and support networks (Fredrickson, 2000; Fredrickson, 2001). In light of the current findings, one way in which a heightened saliency of positive emotions in others may lead to adaptive behaviours and social outcomes may be via the amplification of personal positive affect.

Prior empirical work has demonstrated that heightened sensitivity to positive affective cues is linked with resilient outcomes in socially mediated ways. For example, in the general population, the ability to detect and respond appropriately to affiliative cues can increase the likelihood of more positive social interactions (MacKin et al., 2019) and facilitate an individual’s ability to maintain a wider social network (Aldridge-Waddon et al., 2020; McCrory et al., 2022). In particular, the higher perceived intensity of positive affect is linked with greater perceived trustworthiness and psychological closeness (Engell et al., 2010; Mei et al., 2018; Oosterhof & Todorov, 2009; Van Boven et al., 2010). Assigning greater saliency to more subtle and everyday displays of positive emotions may therefore facilitate prosocial and affiliate behaviours that can help form and maintain supportive relationships over time – a key protective factor following maltreatment experiences (Sperry & Widom, 2013). However, these findings do not mean that all young people with a history of maltreatment are equally likely to experience adaptative changes in how they appraise positive social cues. Changes in social information-processing at a group level may be driven by a subset of individuals more prone to elicit and maintain supportive networks. Therefore, a putative pathway through which social support contributes to resilient psychiatric outcomes (by heightening the saliency of other’s positive emotions) may be more relevant for some individuals than others.

Another theoretical aspect that merits special attention is that the interaction between individual and social factors is not unidirectional (McCrory et al., 2022). The experience of early abuse and neglect shapes cognitive development in ways that may influence an individual’s social experience and the social architecture they build around them. This environment will then exert influence on subsequent cognitive and social development. Experiencing increased salience of positive affect in others may alter an individual’s behaviour that, in turn, elicits different responses from others. Thus, considering the cumulative impact of the transactional processes between individual latent vulnerabilities (i.e. located within the child) and the social environment (i.e. external to, but at times partly created by the child) is an important avenue for future research. Specifically, it will be crucial to delineate the precise mechanisms whereby altered appraisal of positive emotional intensity impacts proximal social interactions and how (using a longitudinal design) this may cumulatively impact, for some children, social and emotional development.

The present findings may lend initial insights into clinically relevant issues. Meta-analytic data shows that a history of childhood abuse and neglect is associated with poorer treatment responses (Nanni et al., 2012; Nelson et al., 2017; Teicher et al., 2022). Although multiple factors are likely to contribute to this, it is well established that being able to form a trusting therapeutic relationship significantly predicts treatment outcomes (Martin et al., 2000; Shirk & Karver, 2003). However, longstanding clinical consensus (Fonagy & Allison, 2014; Martin, 1976) and recent experimental findings (Neil et al., 2022) suggest that building trusting relationships, including those with healthcare professionals, may be more difficult for individuals exposed to invalidating and abusive parental practices. Initial clinical evidence indicates that individuals with a maltreatment history may benefit from psychological interventions that combine cognitive–behavioural treatment principles with a focus on interpersonal factors, the rapport with the therapist, and ‘felt safety’ during sessions (Nemeroff et al., 2003). It is well established that the genuine expression of positive emotions and affirmations, known as ‘positive regard’, is critical for establishing good rapport during treatment and is a predictor of outcomes (Farber & Doolin, 2011) – this may be especially true for individuals with a history of neglect and abuse. Given the critical role that perceived emotional intensity has on trust processing and felt closeness (Engell et al., 2010; Mei et al., 2018; Oosterhof & Todorov, 2009; Van Boven et al., 2010), the preliminary insight from the present study may prove helpful to clinicians as they reflect on their affective communication during rapport building or when providing feedback. Furthermore, the present findings suggest that paying close attention to and strengthening an individual’s social support network (a proposed key mechanisms of change in several evidence-based treatment) (Lipsitz & Markowitz, 2013) may be particularly important for individuals with a history of maltreatment. However, more research is required to establish how current knowledge on positive emotion and social support following maltreatment exposure could be applied in clinical settings.

The results of this study should be considered in the context of a number of limitations. First, the cross-sectional design means we must be tentative in making any mechanistic inference about the associations between the saliency attributed to positive emotions, social support, and mental health outcomes. Although alternative moderated-mediation models were run and showed that exchanging the variable order led to non-significant conditional indirect effects, the current cross-sectional design does not allow us to rule out a possible inverse (or bi-directional) influence between perceived emotional intensity, perceived social support and symptomatology. A longitudinal design, as well as replication within a larger sample, is required to determine the putative directionality of the present model-based findings – i.e. that protective social factors after maltreatment exposure predict adaptive cognitive recalibrations, which, in turn, contribute to resilient mental health outcomes. Second, the present study found that the presence of supportive peers after maltreatment exposure may play a protective role and foster resilient outcomes. However, future studies should examine what factors contribute to the presence of supportive peers in the first place – some of these factors may be individual (e.g. temperament, cognitive style, affective reactivity, interpersonal skills), others may be exogenous (e.g. presence of consistent and supportive carers, socio-economic opportunities, educational settings, degree of community fragmentation and violence). Examining this can help inform and develop psychosocial interventions for those maltreatment-exposed individuals at greater psychiatric risk. Third, because neglect and abuse tend to co-occur for most children and young people exposed to childhood maltreatment, it is beyond the scope of this study to examine if different forms of early adversity have a differential influence on emotional development. Studies with larger sample sizes could explore if one specific maltreatment subtype is more strongly associated with the altered perception of positive emotional intensity. One could speculate that experiences of deprivation and neglect may be more strongly associated with the need to learn from more sparse and inconsistent signals of social rewards. Finally, although we attempted to recruit an ethnically diverse sample, the present study was not designed to examine the potential moderating role of ethnicity and exposure to other forms of adversity, such as racial trauma and discrimination. This is an important issue that future research should address.

This study also has a number of strengths. First, recruiting children and adolescents with substantiated maltreatment experiences and unexposed peers matched for pre-existing characteristics (e.g. socioeconomic status, gender, ethnicity) allows us to address common methodological limitations in the field; such as the conflation of other forms of early adversity (e.g. poverty, illness) with parental maltreatment, studying maltreatment experiences only in the normative or subclinical ranges, and not controlling for the possible confounding role of demographic and cognitive variables (McCrory et al., 2017). Second, we used a robust model-building analytic approach (i.e. mediation and moderation analyses) to examine the association between variables pertaining to different domains. Third, we mitigated potential response biases by implementing multimodal assessments. Past maltreatment experiences were established via institutional records, emotional processing was assessed experimentally, psychological and behavioural functioning included caregivers’ reports, and social support was self-reported. Fourth, the childhood maltreatment literature has tended to privilege the study of implicit processing of affective stimuli and emotional recognition, predominantly negative cues, and their association with psychiatric risk. On the other hand, this study examined the explicit appraisal of others’ emotions. To our knowledge, this has not been previously investigated in the context of maltreatment experience.

In conclusion, this study experimentally examined for the first time if childhood maltreatment experience influences the explicit appraisal of facial emotional intensity. We found that a history of substantiated abuse and neglect is associated with *an increased perceived intensity of positive facial cues*. Exploratory analyses showed that such a response is associated with higher levels of social support and lower mental health symptoms. These findings are consistent with the view that mental health outcomes following maltreatment are likely to emerge through the interaction of emotion processing and a child’s social functioning. The potential clinical implications of the putative protective role of salience attribution to positive social cues, as a contributing factor in achieving favourable treatment outcomes and rapport-building, should be examined in future studies.

## Supplementary Material

Supplementary_Info_Gerin_et_al_EJPT.docx

## Data Availability

The data associated with this study has yet to be made publicly accessible. To discuss access, please contact the Principal Investigator, Professor Eamon McCrory, e.mccrory@ucl.ac.uk.
