# Supplementary material for: Heightened response to positive facial cues as a potential marker of resilience following childhood adversity
Source: Eur J Psychotraumatol. 2024 Feb 6;15(1):2309783. doi: 10.1080/20008066.2024.2309783 (PMC10849006; doi:10.1080/20008066.2024.2309783)
Supplement: Supplementary_Info_Gerin_et_al_EJPT.docx [file ZEPT_A_2309783_SM4202.docx]

**Supplementary Information**

**Heightened response to positive facial cues as a potential marker of resilience following childhood adversity**

Mattia I. Gerin^1,2^, Essi Viding^1^, Louise Neil^1^, Diana J. N. Armbruster-Genc^1^, Ze Freeman^1^, Molly Sharp^1^, Harriet Phillips^1^, Eamon J. McCrory^1,2,*^

*^1^ Division of Psychology and Language Sciences, University College London, London, UK; ^2^ Anna Freud National Centre for Children and Families, London, UK*

*^*^Address Correspondence to this author at Division of Psychology and Language Sciences, University College London, 26 Bedford Way, London WC1H 0AP, UK; Email:* [e.mccrory@ucl.ac.uk](mailto:e.mccrory@ucl.ac.uk).

**Maltreatment Severity and Subtype – Kaufman Scale**

| **Table S1**  Maltreatment Subtype Severity in the MT Group | | | |
| --- | --- | --- | --- |
| Maltreatment Subtype | N | Mean Severity^1^ (1-4) | SD |
| Neglect | 28 | 3.0 | 1.1 |
| Sexual Abuse | 4 | 1.8 | 1.5 |
| Emotional Maltreatment | 38 | 2.3 | 1.1 |
| Home Violence | 36 | 1.6 | 1.0 |
| ^1^Maltreatment severity scores were calculated based on Kaufman et al. (1994) | | | |

The Kaufman’s et al (1994) scale was used to assess the prevalence of maltreatment subtypes and severity among the group of young people with substantiated maltreatment experience (MT Group). This tool examines maltreatment subtypes, including neglect, sexual abuse, emotional maltreatment, and home violence exposure. In this scale, each category of childhood maltreatment is rated from zero to four. The Kappa reliability coefficient for these scales ranged between.73 and .90. The validity of these scales was demonstrated by showing that maltreatment rating was significantly associated with externalising symptoms (r =.57; p<.001) and internalising symptoms (r=.29, p =.03). In terms of inter-rater reliability, the authors noted that “there were no discrepancies between raters that were greater than one scale point”. We provide example from the instrument indicating the most severe rating (i.e. four): Neglect: “Five or more forms of neglect” from a list including poor supervision, not provided routine medical care; Sexual abuse: “ Vaginal or anal intercourse / penetration vaginal or anal intercourse / penetration”; Emotional abuse: “Exposure to parental drug/alcohol abuse, and in addition, there is evidence of extreme parental rejection (e.g. child called unworthy of love, openly rejected, parent threatens to send child away and/or leave child”); Domestic violence: “Exposure to partner physical violence, with weapon (e.g. knife or other object)”.

**Sample’s Ethnic Background Information**

As presented in Table 1, 40.5% in the MT group and 40.6% in the NMT group identified as Caucasian. Furthermore, in the MT group 23.8% identified as Black, 4.8% as Asian; 28.6% as Mixed and 2.4% as ‘Other’. In the NMT group 25.0% identified as Black, 9.4% as Asian; 18.8% as Mixed and 6.3% as ‘Other’.

**Emotion Recognition Accuracy Scores**

A mixed design ANOVA exploring the percentage of accurate recognition of each emotion (fear and happiness) by group (MT and NMT) revealed a significant main effect of emotion type *F*(1,72) = 146.58, *p <* .001, *η_p_^2^ =* .67, such that happy faces were more accurately recognised than fear faces across groups. The main effect of group *F*(1,72) = 1.00, *p =* .32, *η_p_^2^ =* .01, and the interaction effect *F*(1,72) = 0.62, *p =* .43, *η_p_^2^ =* .01 were statistically not significant. That is, across both emotions, the groups did not differ in their ability to correctly recognise facial expressions of fear and happiness. Moreover, the percentage of correctly identified facial expressions for both happiness and fear across all intensity levels (see Table S2) was highly consistent with that reported by Wingenbach et al. (2016), who used the same set of stimuli in a normative group of young adults.

| **Table S2**  *Emotion Recognition Accuracy Score (%) in the maltreated (MT; n=42) and non-maltreated group (NMT; n=32)* | | | |
| --- | --- | --- | --- |
|  |  | MT | NMT |
| *Measures* |  | Mean Percentage (SD) | Mean Percentage (SD) |
| Overall Fear Accuracy | | 57.5 (20.9) | 62.2 (18.0) |
| Low intensity | | 51.1 (25.4) | 56.6 (22.9) |
| Medium intensity | | 58.0 (24.4) | 60.0 (20.8) |
| High intensity | | 63.7 (22.5) | 70.5 (21.6) |
| Overall Happiness Accuracy | | 90.1 (10.8) | 90.8 (10.4) |
| Low intensity | | 79.7 (20.6) | 84.9 (19.4) |
| Medium intensity | | 93.6 (12.5) | 93.8 (10.4) |
| High intensity | | 97.1 (5.9 | 93.9 (9.4) |
| ** p* < .05 | | | |

**Childhood Maltreatment, Social Support, and Perceived Emotional Intensity**

| **Table S3.**  *Cross-sectional moderation model with emotional intensity scores of happy faces as the outcome variable.* | | | |
| --- | --- | --- | --- |
| Predictor | *B*_standardised_ or *d’* | *t* | *p* |
| Maltreatment status | 0.44 | 1.93 | .06 |
| CASSS | -0.5 | -0.39 | .69 |
| Maltreatment status *x* CASSS ** | 0.63 | 2.94 | >.01 |
| **** = *p* < .01.  *Note:* Outcome variable = Mean perceived emotional intensity score for happy faces. Model summary: *R^2^* = .17, *F*(3,67) = 4.13, *p* < .01. The addition of the interaction term significantly increased the variance explained by the model *R^2^-change* = .09, *F*(3,67) = 8.63, *p* < .01. Regression coefficients are expressed as standardised Beta for continuous independent variables and *d’* for categorical independent variables. A heteroscedasticity consistent Huber-White standard error and covariance matrix estimator were used and significance threshold is measured using bootstrapping (n=5000, CI = 95%). *n* = 71 (MT = 40; NMT = 31). *Abbreviations:* CASSS = Child and Adolescent Social Support Scale Frequency Score for the child-reported ‘close friend’ subscale; MT = maltreated group; NMT = non-maltreated group. | | | |

| **Table S4.**  *Effects at different values of the moderator (CASSS) on the association between the independent variable (maltreatment status) and the dependent variable (mean perceived emotional intensity score for happy faces)* | | | |
| --- | --- | --- | --- |
| CASSS Standardised Values (16^th^, 50^th^ and 84^th^ percentile) | Effect _standardised_ | *t* | *p* |
| -0.78 | -0.11 | -0.47 | .64 |
| 0.04 | 0.40* | 2.04 | .05 |
| 1.05 | 1.04** | 3.29 | >.01 |
| ** = p*< .05; **** = *p* < .01.  *n* = 71 (MT = 40; NMT = 31). *Abbreviations:* CASSS = Child and Adolescent Social Support Scale Frequency Score for the child-reported ‘close friend’ subscale. | | | |

**Childhood Maltreatment, Social Support, Perceived Emotional Intensity, and Behavioural and Psychological Functioning**

As depicted in Figure 4 in the manuscript, the moderated-mediation model (based on PROCESS model 7) found that the presence of social support (i.e., CASSS scores ­– the moderator) significantly accounted for the association between maltreatment exposure (i.e., the independent variable) and lower symptoms (i.e., SDQ total score – the dependent variable) via increased emotional intensity scores for happy faces (i.e., the mediator). To assess the possible directionality of this result, five moderated-mediation models were performed by swapping all the variables except for the predictor variable, maltreatment exposure, which was kept constant across models. That is, social support, symptoms severity and emotional intensity scores for happy faces were used in all possible combinations as potential mediators, moderators, or dependent variables.

Across all five moderated-mediation models, the conditional indirect effect was non-significant (significance threshold was measured using bootstrapping, n=5000, CI = 95%; coefficients were standardised): (i) when the SDQ total score was inputted as the dependent variable, CASSS score as the mediator, and emotional intensity scores for happy faces as the moderator, the index of moderated-mediation was non-significant [a_3_ x b = -0.10, 95% CI (-0.27, 0.12)]; (ii) when the emotional intensity scores for happy faces was inputted as the dependent variable, SDQ total score as the mediator, and CASSS score as the moderator, the index of moderated-mediation was non-significant [a_3_ x b = -0.02, 95% CI (-0.17, 0.16)]; (iii) when the emotional intensity scores for happy faces was inputted as the dependent variable, CASSS total score as the mediator, and SDQ total score as the moderator, the index of moderated-mediation was non-significant [a_3_ x b = 0.08, 95% CI (-0.06, 0.25)]; (iv) when CASSS total score was inputted as the dependent variable, SDQ total score as the mediator, and the emotional intensity scores for happy faces as the moderator, the index of moderated-mediation was non-significant [a_3_ x b = -0.02, 95% CI (-0.19, 0.08)]; (v) when CASSS total score was inputted as the dependent variable, the emotional intensity scores for happy faces as the mediator, and SDQ total score as the moderator, the index of moderated-mediation was non-significant [a_3_ x b = -0.01, 95% CI (-0.11, 0.08)].

The fact that across all the alternative moderated-mediation models, the conditional indirect effects were non-significant suggests a possible directionality of the findings. That is, higher levels of social support among children and adolescents with a history of maltreatment may contribute to the increased perception of the emotional intensity of positive affect, which in turn is linked with better mental health symptoms. However, the putative directionality of this finding should be considered with caution, as it requires to be examined in a study with a longitudinal design.

**Childhood Maltreatment, Social Support, Perceived Emotional Intensity, and Behavioural and Psychological Functioning.**

We provide below the results from simple slopes test for the moderation analysis in the moderated-mediation model (i.e. Figure 4, in the main manuscript).

| **Table S5.**  *Effects at different values of the moderator (CASSS) on the association between the independent variable (maltreatment status), the dependent variable (SDQ total score) and the mediator (mean perceived emotional intensity score for happy faces).* | | | |
| --- | --- | --- | --- |
| CASSS Standardised Values (16^th^, 50^th^ and 84^th^ percentile) | Effect _standardised_ | *LLCI* | *ULCI* |
| -0.78 | 0.03 | -0.143 | 0.184 |
| 0.04 | -0.11* | -0.271 | -0.001 |
| 1.05 | -0.29* | -0.556 | -0.067 |
| * = Statistically significant bootstrapped coefficients (n=5,000).  *n* = 71 (MT = 40; NMT = 31). *Abbreviations:* CASSS = Child and Adolescent Social Support Scale Frequency Score for the child-reported ‘close friend’ subscale; SDQ = Strength and Difficulties Questionnaire; LLCI = bootstrapping lower-level confidence interval; ULCI = bootstrapping upper level confidence interval. | | | |
